# Supplementary material for: Layered entrenchment maintains essentiality in the evolution of Form I Rubisco complexes
Source: EMBO J. 2024 Nov 18;44(1):269–80. doi: 10.1038/s44318-024-00311-1 (PMC11696622; doi:10.1038/s44318-024-00311-1)
Supplement: Supplementary file 1 — Appendix [file 44318_2024_311_MOESM1_ESM.pdf]

Layered entrenchment maintains essentiality in the evolution of Form I Rubisco complexes.

Authors:
Luca Schulz, Jan Zarzycki, Wieland Steinchen, Georg K. A. Hochberg*, Tobias J. Erb*

*Correspondence to georg.hochberg@mpi-marburg.mpg.de and toerb@mpi-marburg.mpg.de

Appendix

Table of Contents

Page 2: Appendix Figure S1.
Biochemical characterization of AncL+7 derived variants.
Page 3: Appendix Figure S2.
Mass photometry characterization of AncL+7-based single and double substitution constructs on the trajectory to the AncL+7 R269W E271R L273N.
Page 4: Appendix Figure S3.
Mass photometry characterization of AncL+7 derived variants with XuBP and CABP.
Page 5: Appendix Figure S4.
Mass photometry characterization of AncL+7 R269W E271R L273N without additives and in presence of 3 mM RuBP.
Page 6: Appendix Figure S5.
AncL-based variant inhibition study by XuBP.
Page 7: Appendix Figure S6.
AncL+7-based variant characterization towards XuBP-dependent oligomerization.
Page 8: Appendix Figure S7.
Denaturation of AncL+7-based variants on the trajectory to AncL+7 R269W E271R L273N.
Page 9: Appendix Figure S8.
Hydrogen-deuterium exchange mass spectrometry of AncL+7 LSU and LSU+CABP.
Page 10: Appendix Table S1.
Kinetic characterization of AncL+7-based variants.
Page 11: Appendix Table S2.
List of enzyme variants used in this study.
Page 12: Appendix Table S3.
Crystallography data for AncL+7 REL and AncL+7 REL with AncSSU.
Page 14: Appendix Table S4.
List of primers used in this study.
Page 15: Appendix Table S5.
List of plasmids used in this study.
Page 16: Appendix Table S6.
List of strains used in this study.

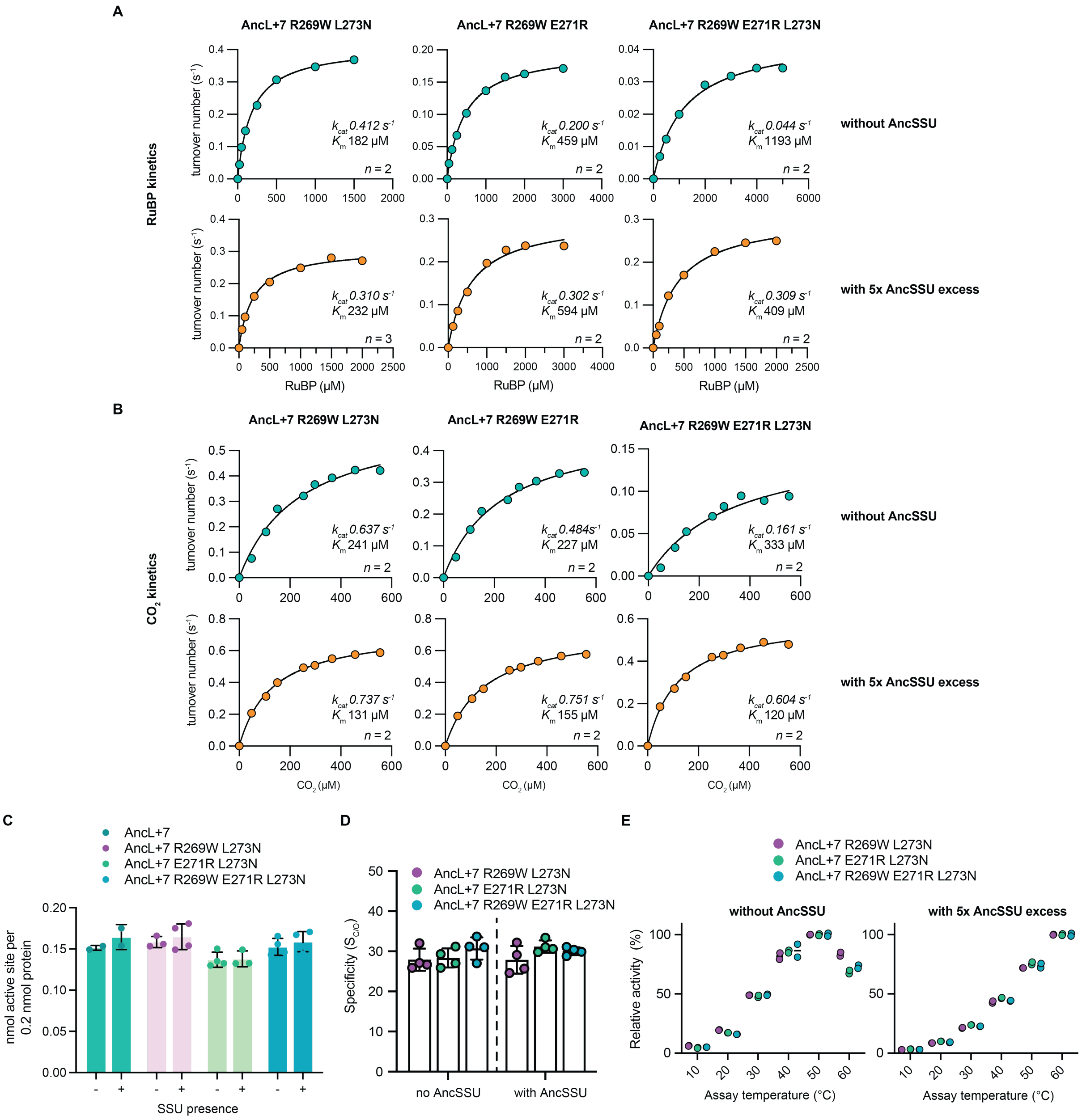

Appendix Figure S1. Biochemical characterization of AncL+7 derived variants. (**A**) RuBP kinetics of AncL+7-based variants AncL+7 R269W L273N, AncL+7 R269W E271R, and R269W E271R L273N measured without (teal) or with (orange) a 5x AncSSU excess. *N* = indicated. k_{cat} and K_m are listed in the plots (see Appendix Table S1). (**B**) CO_2 kinetics of AncL+7-based variants AncL+7 R269W L273N, AncL+7 R269W E271R, and R269W E271R L273N measured without (teal) or with (orange) a 5x AncSSU excess. *N* = 2. k_{cat} and K_m are listed in the plots. (**C**) Relative CABP binding of relevant Rubisco variants with or without presence of a 5-fold AncSSU excess. Mean of measurements depicted with error bars showing the standard deviation. Individual data points are shown. (**D**) Specificity ($S_{C/O}$) of relevant Rubisco variants with or without presence of a 5-fold AncSSU excess. Mean of *N* = 4 measurements depicted with error bars showing the standard deviation. Individual data points are shown. (**E**) Relative activity of relevant Rubisco variants at varying temperatures without AncSSU (left) and with a 5-fold AncSSU excess (right). Activity is relative to the highest measured activity of the variant in question, for either the dataset with or without AncSSU. Mean of *N* = 2 measurements depicted with individual data points shown.

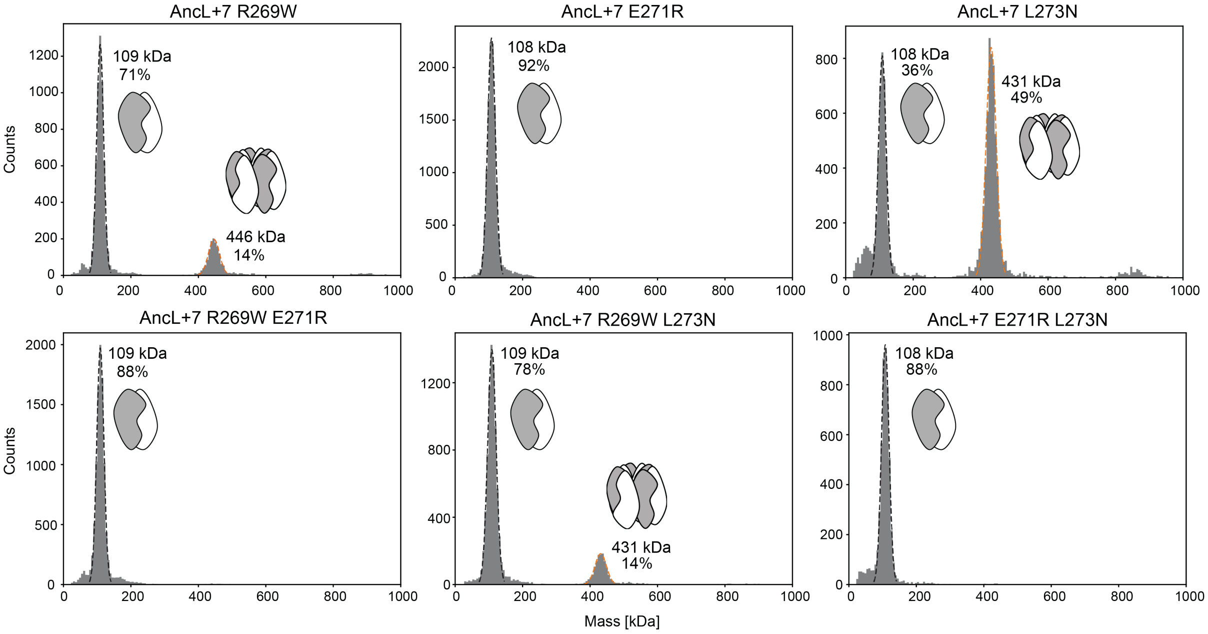

Appendix Figure S2. Mass photometry characterization of AncL+7-based single and double substitution constructs on the trajectory to the AncL+7 R269W E271R L273N. Single substitution constructs in top row, double substitution constructs in bottom row. Percentages indicate integrated counts relative to all counts of the measurement.

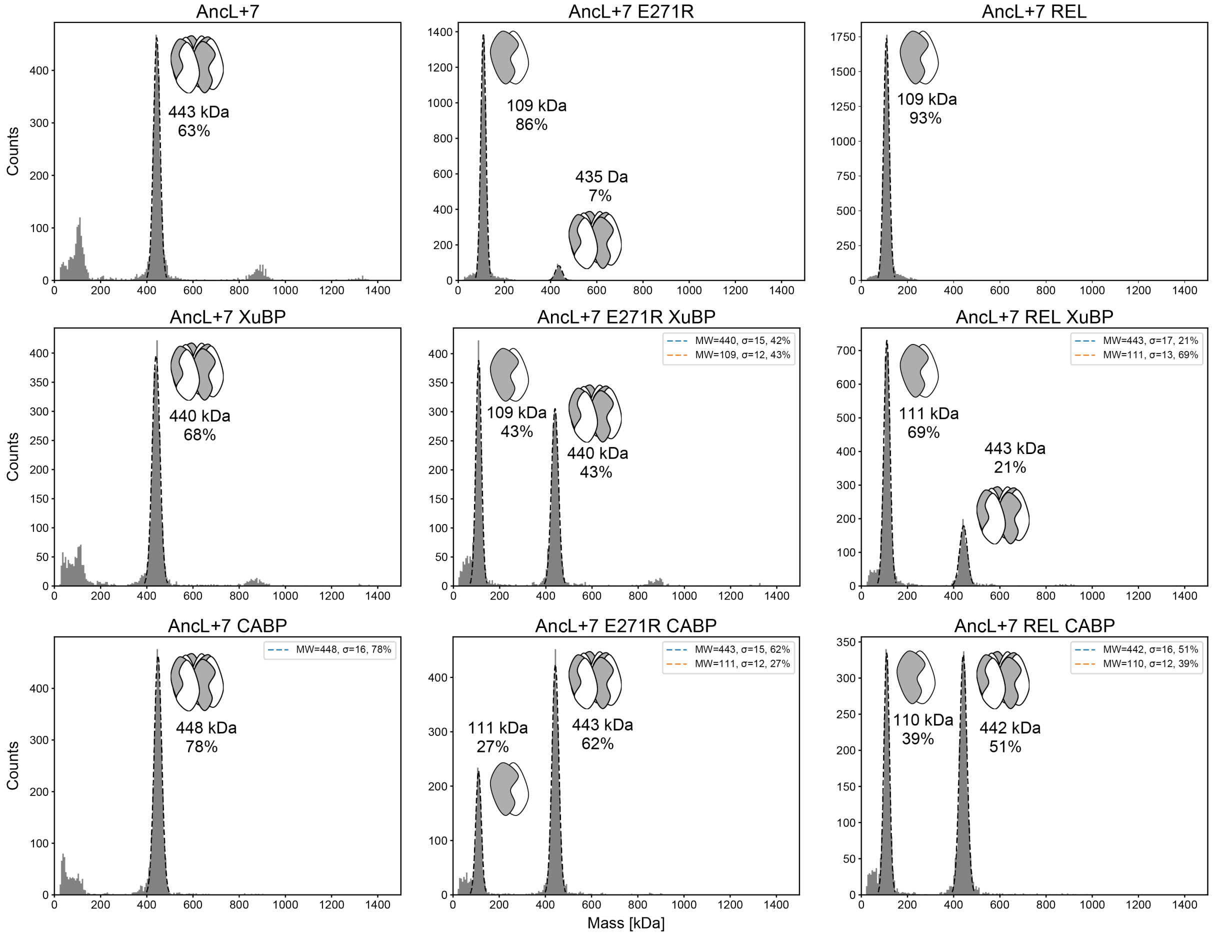

Appendix Figure S3. Mass photometry characterization of AncL+7, AncL+7 E271R, and AncL+7 R269W E271R L273N (denoted as AncL+7 REL) alone, with xylulose-1,5-bisphosphate (XuBP, 1 mM) and carboxyarabinitol-bisphosphate (CABP, 0.1 mM). First row contains measurements in isolation, second row contains measurements with XuBP, third row contains measurements with CABP. Percentages indicate integrated counts relative to all counts of the measurement.

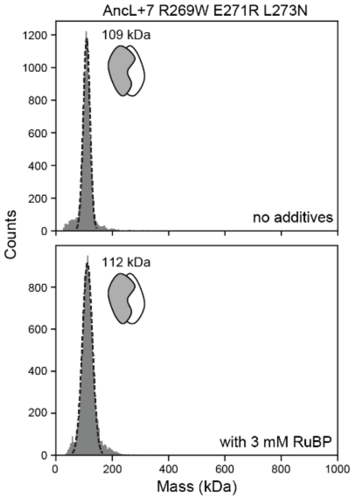

Appendix Figure S4. Mass photometry characterization of AncL+7 R269W E271R L273N without additives and in presence of 3 mM RuBP. Top shows measurement in isolation, bottom shows measurements with RuBP. Protein was pre-incubated at 20 µM concentration in presence of 3 mM RuBP for >30 min prior to crash dilution to 500 nM protein concentration and subsequent measurement at 50 nM protein concentration. Theoretical mass of a dimer is 107 kDa and of an octamer 428 kDa.

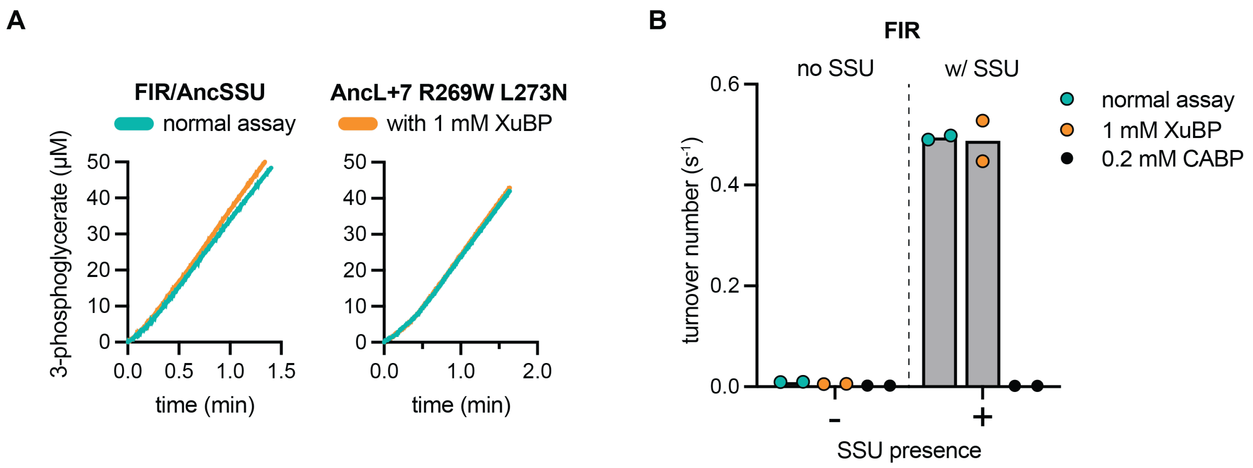

Appendix Figure S5. AncL-based variants are not inhibited by XuBP. (**A**) Unchanged 3-phosphoglycerate production of FIR construct or AncL+7 R269W L273N either in isolation or in presence of 1 mM XuBP during activation and the assay. Mean of *N* = 2 measurements depicted. (**B**) Absolute turnover number (s^{-1}) of FIR construct shows SSU dependence and unchanged turnover in presence of 1 mM XuBP, while the addition of 0.2 mM CABP fully inhibits the enzyme. Mean of *N* = 2 measurements depicted. Individual data points are shown.

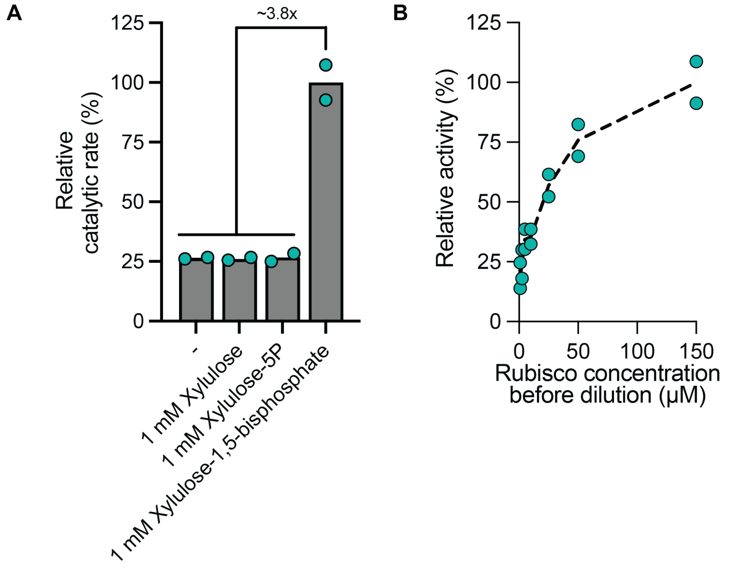

Appendix Figure S6. AncL+7-based variant characterization towards XuBP-dependent oligomerization. **(A)** Activity of AncL+7 R269W, E271R, L273N only increases in presence of 1 mM xylulose-1,5-bisphosphate and is unresponsive to the addition of mono- or non-phosphorylated xylulose. Mean of $N = 2$ measurements depicted. Individual data points are shown. **(B)** Activity of AncL+7 E271R crash diluted into assay mixture (normalized to the same concentration in the assay) from different concentrations shows a positive correlation with increasing pre-dilution concentrations. Activity is given relative to the measurements of 150 µM Rubisco pre-dilution. Mean of $N = 2$ measurements depicted. Individual data points are shown, dashed line is shown to help guide the reader.

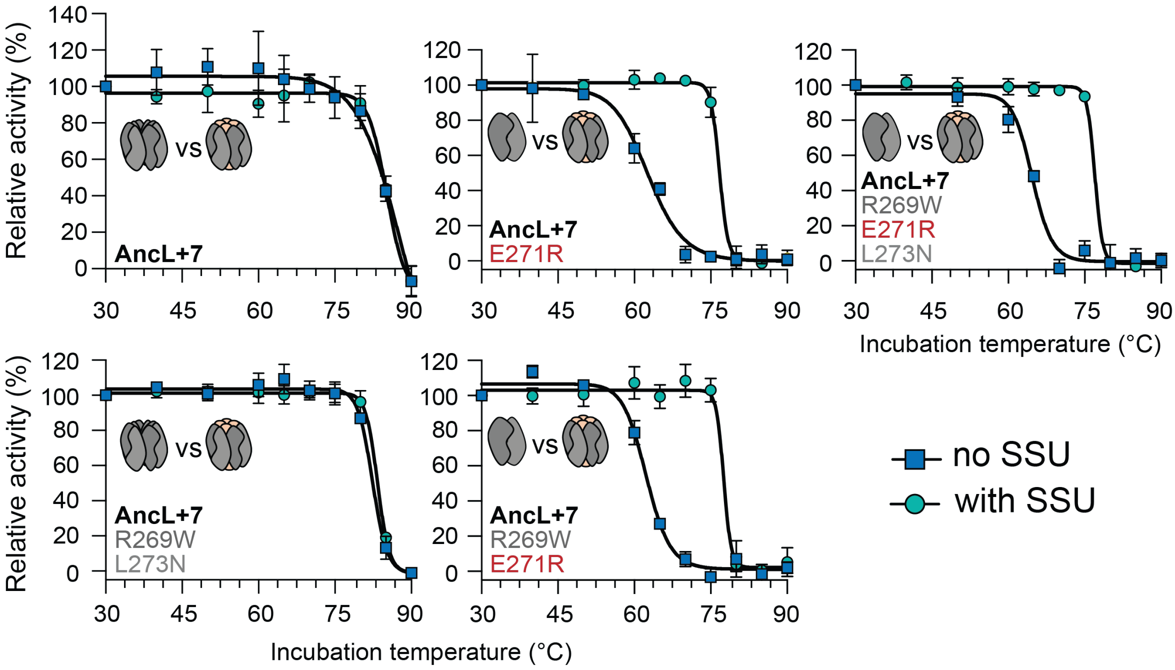

Appendix Figure S7. Denaturation of AncL+7-based variants on the trajectory to AncL+7 R269W E271R L273N indicate that variants which contain E271R (highlighted in red) and thus disassemble into dimers in absence of AncSSU are destabilized to thermal denaturation (see cartoon schemes for inferred oligomeric state). Variants were incubated at indicated temperatures for 1 hour prior to cooling down and measuring remaining activity at 25 °C. A 5-fold AncSSU excess was used for incubations in presence of SSU. Mean of N = 3 measurements depicted with error bars showing the standard deviation.

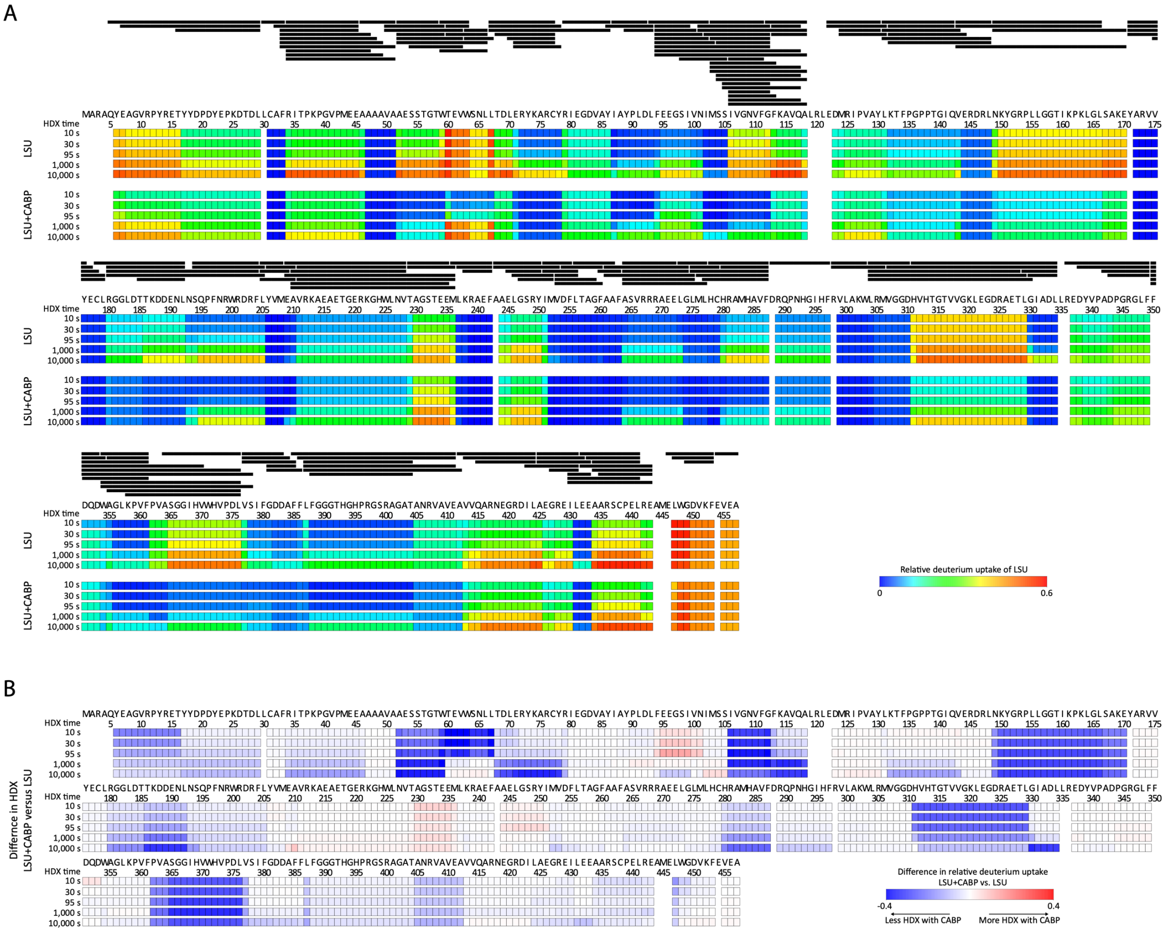

Appendix Figure S8. Hydrogen-deuterium exchange mass spectrometry of AncL+7 LSU and LSU+CABP. (**A**) Relative deuterium uptake of Rubisco's large subunit in LSU and LSU+CABP conditions. Detected peptides are indicated as black bars above the measurements. (**B**) Difference in hydrogen-deuterium exchange between LSU+CABP versus just the LSU.

Appendix Table S1 | Kinetic characterization of AncL+7-based variants

variant	k_{cat} (CO_2) (s^{-1})	K_m (CO_2) (µM)	k_{cat} (RuBP) (s^{-1})	K_m(RuBP) (µM)
Fiber-interface reversion	Unstable fit	Unstable fit	n.d.	n.d.
Fiber-interface reversion +AncSSU	1.58 ± 0.08 (2)	61 ± 7 (2)	n.d.	n.d.
AncL+7	n.d.	n.d.	0.42 ± 0.01 (2)	132 ± 14 (2)
AncL+7 + AncSSU	n.d.	n.d.	0.26 ± 0.02 (2)	402 ± 90 (2)
AncL+7 G158C L192I	n.d.	n.d.	0.05 ± 0.00 (2)	435 ± 102 (2)
AncL+7 G158C L192I + AncSSU	n.d.	n.d.	0.13 ± 0.00 (2)	161 ± 16 (2)
AncL+7 R269W L273N	0.64 ± 0.07 (2)	241 ± 65 (2)	0.41 ± 0.01 (2)	182 ± 19 (2)
AncL+7 R269W L273N + AncSSU	0.74 ± 0.04 (2)	131 ± 20 (2)	0.31 ± 0.01 (2)	232 ± 24 (2)
AncL+7 R269W E271R	0.48 ± 0.05 (2)	227 ± 58 (2)	0.20 ± 0.01 (2)	459 ± 63 (2)
AncL+7 R269W E271R + AncSSU	0.75 ± 0.03 (2)	155 ± 18 (2)	0.30 ± 0.02 (2)	594 ± 128 (2)
AncL+7 R269W E271R L273N	0.16 ± 0.04 (2)	333 ± 177 (2)	0.04 ± 0.00 (2)	1193 ± 217 (2)
AncL+7 R269W E271R L273N + AncSSU	0.60 ± 0.03 (2)	120 ± 21 (2)	0.31 ± 0.02 (2)	409 ± 78 (2)

Values are mean ± 95% confidence intervals with the number of technical replicates (*N*) indicated in parentheses (n.d. = not determined). Values for k_{cat} (CO_2) and k_{cat} (RuBP) diverge because they were measured with different assays (radioactive CO_2 incorporation versus coupled enzymatic assays, respectively). Measurements in presence of AncSSU were conducted with a five-fold AncSSU excess, except for the fiber-interface reversion construct measurements, which were conducted using a ten-fold AncSSU excess.

Appendix Table S2 | List of enzyme variants used in this study.

variant name	parent variant	relevant phenotype	substitutions from parent	ref.
AncL	AncL	Ancestral Rubisco large subunit that cannot bind AncSSU. Created via ancestral sequence reconstruction.	None	(Schulz *et al*, 2022a)
AncL+7	AncL	Ancestral Rubisco large subunit engineered to bind AncSSU by introducing 7 historical substitutions	Q66L, E142Q, V150K, N382D, A414V, A415Q, T418N	(Schulz *et al*, 2022a)
AncLS	AncLS	Ancestral Rubisco large subunit that can bind AncSSU and is strictly dependent on it. Created via ancestral sequence reconstruction.	None	(Schulz *et al*, 2022a)
Fiber interface reversion (FIR)	AncLS	Rubisco large subunit based on AncLS that contains 14 reversions towards AncL, which were introduced to restore solubility in the absence of AncSSU. Substitutions were identified by being in proximity to the polymerization interface between AncLS octamers. Notably, this variant is not active without AncSSU.	R3K, Q5A, E61T, Q66L, H393L, R397W, S399N, R400A, V410L, V432K, S437W, C438S, E443A, L447T	(Schulz *et al*, 2022a)
AncL+7 S437W	AncL+7	Ancestral Rubisco large subunit engineered to bind AncSSU by introducing 7 historical substitutions. Additionally contains a single serine to tryptophan substitution that causes the Rubisco large subunit octamer to lose solubility in absence of AncSSU.	S437W	(Schulz *et al*, 2022a)
AncSSU	AncSSU	Ancestral Rubisco small subunit derived from ancestral sequence reconstruction.	None	(Schulz *et al*, 2022a)
AncL+7 R269W	AncL+7	AncL+7 (see above) with a substitution aimed at disrupting octamer formation.	R269W	this work
AncL+7 E271R	AncL+7	AncL+7 (see above) with a substitution aimed at disrupting octamer formation. This single substitution variant is already impaired in its ability to form octamers at assay conditions, as evidenced by mass photometry and a reduction in catalytic activity.	E271R	this work
AncL+7 L273N	AncL+7	AncL+7 (see above) with a substitution aimed at disrupting octamer formation.	L273N	this work
AncL+7 R269W E271R	AncL+7	AncL+7 (see above) with two substitutions aimed at disrupting octamer formation. This double substitution variant is already impaired in its ability to form octamers at assay conditions, as evidenced by mass photometry and a reduction in catalytic activity.	R269W, E271R	this work
AncL+7 R269W L273N	AncL+7	AncL+7 (see above) with two substitutions aimed at disrupting octamer formation.	R269W, L273N	this work
AncL+7 E271R L273N	AncL+7	AncL+7 (see above) with two substitutions aimed at disrupting octamer formation. This double substitution variant is already impaired in its ability to form octamers at assay conditions, as evidenced by mass photometry and a reduction in catalytic activity.	E271R, L273N	this work
AncL+7 REL	AncL+7	AncL+7 (see above) with three substitutions aimed at disrupting octamer formation. This triple substitution variant is heavily impared in its ability to form octamers at assay conditions and loses most of its catalytic activity.	R269W, E271R, L273N	this work
AncL+7 R11K	AncL+7	AncL+7 (see above) with a substitution aimed at disrupting catalytic activity in the absence of AncSSU.	R11K	this work
AncL+7 P12E	AncL+7	AncL+7 (see above) with a substitution aimed at disrupting catalytic activity in the absence of AncSSU.	P12E	this work
AncL+7 E15Q	AncL+7	AncL+7 (see above) with a substitution aimed at disrupting catalytic activity in the absence of AncSSU.	E15Q	this work
AncL+7 N65D	AncL+7	AncL+7 (see above) with a substitution aimed at disrupting catalytic activity in the absence of AncSSU. This variant has reduced catalytic activity in absence of AncSSU.	N65D	this work
AncL+7 G158C	AncL+7	AncL+7 (see above) with a substitution aimed at disrupting catalytic activity in the absence of AncSSU. This variant has reduced catalytic activity in absence of AncSSU.	G158C	this work
AncL+7 L192I	AncL+7	AncL+7 (see above) with a substitution aimed at disrupting catalytic activity in the absence of AncSSU. This variant has reduced catalytic activity in absence of AncSSU.	L192I	this work
AncL+7 V312I	AncL+7	AncL+7 (see above) with a substitution aimed at disrupting catalytic activity in the absence of AncSSU.	V312I	this work
AncL+7 E327S	AncL+7	AncL+7 (see above) with a substitution aimed at disrupting catalytic activity in the absence of AncSSU.	E327S	this work
AncL+7 V373M	AncL+7	AncL+7 (see above) with a substitution aimed at disrupting catalytic activity in the absence of AncSSU.	V373M	this work
AncL+7 D375E	AncL+7	AncL+7 (see above) with a substitution aimed at disrupting catalytic activity in the absence of AncSSU.	D375E	this work
FIR C170G	FIR	Fiber interface reversion (FIR)-based mutant that contains a C170G substitution. C170G is the reversion of C158G, which was introduced to restore catalytic activity in the fiber interface reversion construct. The difference in numbering stems from insertions in the parent variants.	C170G	this work
FIR I204L	FIR	Fiber interface reversion (FIR)-based mutant that contains a I204L substitution. I204L is the reversion of L192I, which was introduced to restore catalytic activity in the fiber interface reversion construct. The difference in numbering stems from insertions in the parent variants.	I204L	this work

FIR catrev	FIR	Fiber interface reversion (FIR)-based mutant that contains C170G and I204L substitutions. C170G and I204L are the reversions of C158G and L192I, which were introduced to restore catalytic activity in the fiber interface reversion construct. The difference in numbering stems from insertions in the parent variants.	C170G, I204L	this work

Appendix Table S3 | Crystallography data for AncL+7 REL and AncL+7 REL with AncSSU

Rubisco variant	AncL+7 REL<br><br>(PDB 8QMV)	AncL+7 REL<br>AncSSU<br>(PDB 8QMW)
**Data collection**		
Beamline	PETRA III P14	PETRA III P14
Wavelength (Å)	0.6888	0.6888
Space Group	$C\ 2\ 2\ 2_1$	$C\ 1\ 2\ 1$
Unit cell dimensions		
a, b, c (Å)	121.79, 203.80, 147.37	206.01, 106.44, 108.49
α, β, γ (°)	90.00, 90.00, 90.00	90.00, 113.09, 90.00
Resolution (Å)	24.97 - 1.85 (1.916 - 1.850)	24.88 - 1.75 (1.813 - 1.750)
Unique reflections	155284 (15351)	213396 (21212)
Multiplicity	13.9 (12.6)	7.2 (7.3)
Completeness (%)	99.9 (99.6)	98.6 (98.4)
$I / \sigma I$	16.0 (2.2)	11.1 (2.7)
R_{merge}	0.113 (1.04)	0.124 (0.653)
R_{pim}	0.031 (0.302)	0.049 (0.258)
$CC_{1/2}$	0.999 (0.762)	0.997 (0.834)
**Refinement**		
R_{work} / R_{free}	0.1607 / 0.1739	0.1448 / 0.1745
RMS bonds	0.003	0.013
RMS angles	0.666	1.148
Ramachandran		
favored (%)	97.30	96.56
allowed (%)	2.70	3.44
outliers (%)	0.00	0.00
Number of atoms	15421	19717
Protein	14180	17595
Ligands	88	107
Solvent	1153	2015
Average B-factor	28.46	18.47
Protein	28.05	17.46
Ligands	23.02	15.25
Solvent	33.84	27.42

Values in parentheses are for highest-resolution shell.

Appendix Table S4 | List of primers used in this study.

primer	target	sequence	purpose
pET28b_lin_fw	pET28b	GGTATATCTCCTTCTTAAAGTTAAAC	Linearization
pET28b_lin_rv	pET28b	CACCACCACCACCACCACTGAG	Linearization
pET16b_lin_fw	pET16b	ATGACGACCTTCGATATGGCC	Linearization
pET16b_lin_rv	pET16b	GGATCCGGCTGCTAACAAAG	Linearization
AncL7_REL_WRN_fw	AncL+7	GTtggGCAcgcGAAaacGGTCTCATGCTGCATTGC	Mutagenesis
AncL7_REL_WRN_rev	AncL+7	GCCGCACCGAAGCGAAAGCTG	Mutagenesis
AncL7_R269W_fw	AncL+7	GTtggGCAGAGGAATTAGGTCTCATGCTGCATTG	Mutagenesis
AncL7_E271R_fw	AncL+7	GTCGCGCAcgcGAATTAGGTCTCATGCTGCATTG	Mutagenesis
AncL7_L273N_fw	AncL+7	GTCGCGCAGAGGAAaacGGTCTCATGCTGCATTG	Mutagenesis
AncL7_R269W_E271R_fw	AncL+7	GTtggGCAcgcGAATTAGGTCTCATGCTGCATTG	Mutagenesis
AncL7_R269W_L273N_fw	AncL+7	GTtggGCAGAGGAAaacGGTCTCATGCTGCATTG	Mutagenesis
AncL7_E271R_L273N_fw	AncL+7	GTCGCGCAcgcGAAaacGGTCTCATGCTGCATTG	Mutagenesis
FIR_WRN_REL_fw	FIR_construct	ggagctgGGAATGCTGCTGCATTTAC	Mutagenesis
FIR_WRN_REL_rev	FIR_construct	tcgcagcgCTTACTCAAGCTCGTAAAAG	Mutagenesis
AncL7_R11K_fw	AncL+7	AGCGGGTGTTaaaCCGTATCGCGAAACC	Mutagenesis
AncL7_R11K_rev	AncL+7	TCATATTGGGCCCGCGCC	Mutagenesis
AncL7_P12E_fw	AncL+7	GGGTGTTCGCgaaTATCGCGAAACCTATTATGATC	Mutagenesis
AncL7_P12E_rev	AncL+7	GCTTCATATTGGGCCCGC	Mutagenesis
AncL7_E15Q_fw	AncL+7	CCCGTATCGCcagACCTATTATGATC	Mutagenesis
AncL7_E15Q_rev	AncL+7	CGAACACCCGCTTCATAT	Mutagenesis
AncL7_N65D_fw	AncL+7	GGTTTGGTCTgacCTGTTAACCGACCTTG	Mutagenesis
AncL7_N65D_rev	AncL+7	TCGGTCCAGGTGCCTGTC	Mutagenesis
AncL7_G158C_fw	AncL+7	GCTTCTCGGCtgtACTATCAAAC	Mutagenesis
AncL7_G158C_rev	AncL+7	GGACGACCGTATTTGTTC	Mutagenesis
AncL7_L192I_fw	AncL+7	CGATGAAAATatcAACTCGCAACCG	Mutagenesis
AncL7_L192I_rev	AncL+7	TCTTTGGTCGTGTCCAGG	Mutagenesis
AncL7_V312I_fw	AncL+7	CGGTGACCATatcCACACCGGAA	Mutagenesis
AncL7_V312I_rev	AncL+7	CCAACCATGCGGAGCCATTTG	Mutagenesis
AncL7_E327S_fw	AncL+7	GGATCGCGCGagcACACTGGGTA	Mutagenesis
AncL7_E327S_rev	AncL+7	CCCTCCAGTTTACCGACG	Mutagenesis
AncL7_V373M_fw	AncL+7	CGTGTGGCACatgCCTGATCTGG	Mutagenesis
AncL7_V373M_rev	AncL+7	TGGATACCGCCGGACGCA	Mutagenesis
AncL7_D375E_fw	AncL+7	GCACGTGCCTgaaCTGGTATCTATC	Mutagenesis
AncL7_D375E_rev	AncL+7	CACACGTGGATACCGCCG	Mutagenesis
FIR_C170g_fw	FIR_construct	GCTTTTAGGGggcACTATTAAAC	Mutagenesis
FIR_C170g_rev	FIR_construct	GGGCGCCCGTACTT	Mutagenesis
FIR_I204L_fw	FIR_construct	CGATGAAAATctgAATTCGCAGCC	Mutagenesis
FIR_I204L_rev	FIR_construct	TCTTTGGTGAAATCCAG	Mutagenesis

Lowercase letters denote point of mutagenesis.

Appendix Table S5 | List of plasmids used in this study.

plasmid	relevant features	source or reference
pET16b	*E. coli* expression vector, T7 promoter, Amp^R, *lacI*, N-terminal His_{10}-tag	Merck Chemicals
pET28b	*E. coli* expression vector, T7 promoter, Kan^R, *lacI*, C-terminal His_6-tag	Merck Chemicals
pET16b-AncL+7	*E. coli* expression vector, T7 promoter, Amp^R, *lacI*, N-terminal His_{10}-tag	(Schulz *et al*, 2022a)
pET16b-fiber interface reversion	*E. coli* expression vector, T7 promoter, Amp^R, *lacI*, N-terminal His_{10}-tag	(Schulz *et al*, 2022a)
pET28b-AncSSU	*E. coli* expression vector, T7 promoter, Kan^R, *lacI*, C-terminal His_6-tag	(Schulz *et al*, 2022a)

Genes cloned into pET16b vectors were cloned in-frame with an N-terminal His-tag encoding sequence on the LSU. Given list does not include point mutant variant-encoding plasmids derived from the plasmid above via Q5 mutagenesis.

Appendix Table S6 | List of strains used in this study.

strain	genotype	source or reference
E. coli NEB Turbo	F´ *proA*$^{+}$*B*$^{+}$ *lacI*^{q} ∆ *lacZ* M15/*fhuA2* ∆(*lac-proAB*) *glnV gal R*(*zgb-210::Tn10*)Tet^{S} *endA1 thi-1* ∆(*hsdS-mcrB*)5	New England Biolabs
E. coli BL21 (DE3)	*E. coli* str. B F^{-} *ompT gal dcm lon hsdS*$_{B}$($r_B^- m_B^-$) λ(DE3 [*lacI lacUV5-T7p07 ind1 sam7 nin5*]) [*malB*$^{+}$]$_{K-12}$(λ^{S})	Thermo Fisher Scientific
E. coli ArcticExpress (DE3)	*E. coli* str. B F^{-} *ompT hsdS*($r_B^- m_B^-$) *dcm*$^{+}$ Tet^{r} *gal* λ(DE3) *endA* Hte [*cpn10 cpn60* Gent^{r}]	Agilent Technologies
